# Correction: Functional drug carriers formed by RGD-modified β-CD-HPG for the delivery of docetaxel for targeted inhibition of nasopharyngeal carcinoma cells

**DOI:** 10.1039/d2ra90065c

**Published:** 2022-07-01

**Authors:** Lingling Xu, Chuan Zhou, Fan Wang, Huiqin Liu, Guangyuan Dong, Siyi Zhang, Tao Liu

**Affiliations:** Department of Otolaryngology-Head and Neck Surgery, Guangdong Provincial People's Hospital, Guangdong Academy of Medical Sciences Guangzhou Guangdong 510080 China szhang555@hotmail.com taoliu18@126.com; The Second School of Clinical Medicine, Southern Medical University Guangzhou Guangdong 510515 China; Shantou University Medical College Shantou 515063 PR China; Key Laboratory of Biomaterials of Guangdong Higher Education Institutes, Department of Biomedical Engineering, Jinan University Guangzhou 510632 China

## Abstract

Correction for ‘Functional drug carriers formed by RGD-modified β-CD-HPG for the delivery of docetaxel for targeted inhibition of nasopharyngeal carcinoma cells’ by Lingling Xu *et al.*, *RSC Adv.*, 2022, **12**, 18004–18011. https://doi.org/10.1039/D2RA02301F.

The authors regret that the equal author contributions and corresponding author indicators were incorrectly shown in the original manuscript. The corrected indicators are as shown in this correction notice.

The Royal Society of Chemistry apologises for these errors and any consequent inconvenience to authors and readers.

## Supplementary Material

